# Active glomerular inflammation versus chronicity and fibrosis: the role of targeted therapies in IgA nephropathy

**DOI:** 10.1093/ndt/gfaf059

**Published:** 2025-03-25

**Authors:** Jai Radhakrishnan, Richard A Lafayette

**Affiliations:** Division of Nephrology, Columbia University, New York, NY, USA; Stanford Glomerular Disease Center, Stanford University, Stanford, CA, USA

Globally, immunoglobulin A nephropathy (IgAN) is the most common form of primary glomerulonephritis and a leading cause of kidney failure [1, 2]. IgAN is characterized by the deposition of pathogenic immune complexes containing galactose-deficient immunoglobulin A1 (Gd-IgA1) in the kidney, predominantly in the glomerular mesangium [1]. The widely accepted pathogenesis of primary IgAN consists of multiple sequential hits: elevated production of Gd-IgA1 from mucosa-associated lymphoid tissue (hit 1); production of anti–Gd-IgA1 autoantibodies (hit 2); formation of circulating immune complexes containing Gd-IgA1 and anti–Gd-IgA1 autoantibodies (hit 3); and deposition of immune complexes in the mesangium (hit 4), leading to glomerular inflammation and injury [3].

**Figure 1:**
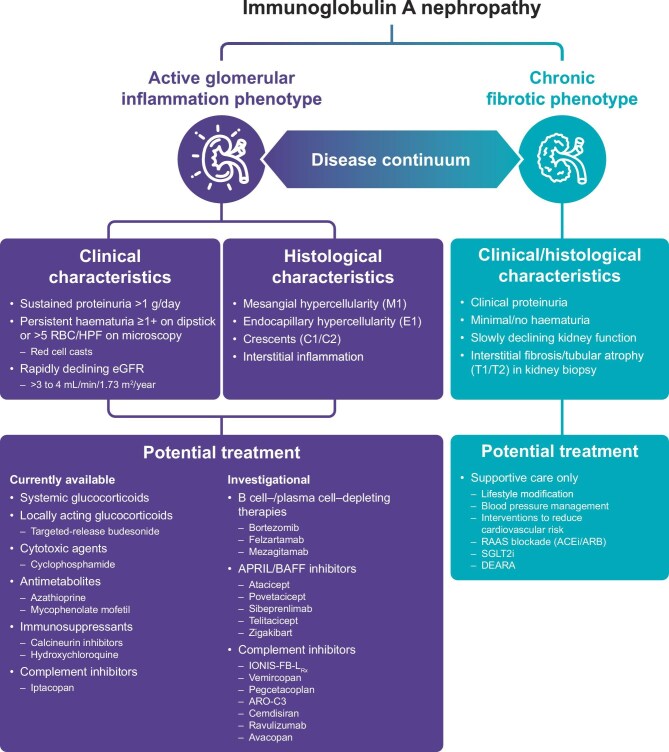
Active glomerular inflammation versus chronic fibrotic phenotype in IgAN and potential therapeutic strategies [2–7]. The extremes of the IgAN histological spectrum consist of an inflammatory glomerular phenotype and a chronic fibrotic phenotype, which can be described by a combination of clinical and histological disease markers. Current approaches for the treatment of glomerular inflammation include systemic glucocorticoids, targeted-release budesonide, cytotoxic agents, antimetabolites, immunosuppressants and the recently approved complement factor B inhibitor, iptacopan. Several anti-inflammatory treatments with the potential to reduce glomerular inflammation in IgAN are in clinical development. ACEi, angiotensin-converting enzyme inhibitor; ARB, angiotensin II receptor blocker; DEARA, dual endothelin angiotensin receptor antagonist; HPF, high-powered field; RAAS, renin–angiotensin–aldosterone system; RBC, red blood cell; SGLT2i, sodium-glucose cotransporter-2 inhibitor.

It is well recognized that IgAN is a heterogeneous disease, with marked variability in clinical presentation, histopathology, disease course and treatment response [1, 2]. Although all patients with IgAN have glomerular Gd-IgA1 immune complex deposition, other histological components on kidney biopsy are highly variable [1, 4]. It is clear that the extremes of the IgAN histological spectrum encompass an ‘active glomerular inflammation’ phenotype and a ‘chronic fibrotic’ phenotype, with many patients exhibiting variable amounts of glomerular inflammation and fibrosis in the middle of the spectrum (Fig. 1) [2–7]. Notably, active glomerular inflammation may resolve and reemerge, spontaneously or following therapy, throughout a patient's life.

Although specific criteria defining the phenotype of active glomerular inflammation in IgAN are not yet established, a combination of clinical and kidney biopsy parameters is helpful. Histological features supportive of active glomerular inflammation include the presence of mesangial hypercellularity (M1), endocapillary hypercellularity (E1) or crescents (C1/C2), as per the Oxford Classification of IgAN, and interstitial inflammation on kidney biopsy [4, 7]. Based on the authors’ experience, clinical findings likely indicative of active glomerular inflammation include sustained proteinuria >1 g/day, persistent haematuria (particularly when seen with red cell casts) and rapidly declining kidney function. Clinically, it may not be easy to distinguish between the active inflammatory and chronic fibrotic phenotypes since, for example, substantial proteinuria and a reduced estimated glomerular filtration rate (eGFR) may occur at all stages of IgAN. Conversely, patients with E1 or C1 lesions on kidney biopsy may present with proteinuria levels <1 g/day [8]. Alone, sustained proteinuria >1 g/day is not indicative of the active inflammatory phenotype, as patients with glomerular scars, especially focal segmental scars, may have heavy proteinuria [9]. Similarly, haematuria can result from structural defects in glomerulosclerotic lesions, including the presence of gaps, shunts or thinning in the glomerular basement membrane [10, 11]. Importantly, all clinical and histological findings in each patient should be considered together to identify this phenotype. In contrast, the chronic fibrotic phenotype, although associated with similar levels of proteinuria and a slow eGFR decline, is typically indicated by interstitial fibrosis/tubular atrophy (T1/T2), which is associated with higher CKD stage and is the strongest predictor of an adverse kidney outcome in a biopsy [4, 7, 12].

Inflammation of the glomerulus in IgAN arises from the interplay of multiple factors. Glomerular immune complex deposits induce mesangial cells to proliferate and overproduce extracellular matrix, as well as inflammatory and pro-fibrotic cytokines and chemokines [1]. The mesangial cell-derived factors, including tumour necrosis factor alpha (TNF-α), transforming growth factor beta (TGF-β), interleukin-6 (IL-6) and angiotensin II, then contribute to proteinuria and tubulointerstitial injury by altering glomerular permeability via cross-talk with, and damage to, podocytes and tubular epithelial cells [1, 13]. Immune complexes also activate complement, which may not be limited to the kidneys but also occur systemically in the fluid phase, with the glomeruli being the predominant site of injury [1, 13]. Complement activation recruits immune cells to sites of inflammation, predominantly via the C3a and C5a anaphylatoxins, and leads to direct damage to glomerular cells by formation of the membrane attack complex [14]. The alternative and lectin pathways, specifically, are believed to play major roles in IgAN pathophysiology, as demonstrated by the glomerular deposition of complement proteins associated with these pathways [13]. Although the mechanism of immune complex–mediated activation of the alternative and lectin pathways in IgAN remains poorly understood, *in vitro* studies suggest that Gd-IgA1 immune complexes can act as activation surfaces for these pathways [13]. In addition, evidence of complement activation has been associated with more severe histologic lesions in IgAN, further supporting the role of complement in the development of active glomerular inflammation. For example, glomerular deposition of complement proteins such as C3, C4d, MBL and C5b9 have been correlated with histologic markers of IgAN severity and damage [13].

Initial disease management for patients with IgAN has largely been based on optimized supportive care, as recommended by the Kidney Disease: Improving Global Outcomes (KDIGO) 2021 guideline, including blood pressure management, renin–angiotensin–aldosterone system inhibitors, lifestyle modification and cardiovascular risk reduction [2]. The approval of sodium-glucose cotransporter-2 inhibitors and sparsentan (a dual endothelin A receptor antagonist and angiotensin II receptor blocker) represent additional options for therapy aimed at reducing progression of the chronic fibrotic phenotype [3, 6].

Clinical or histological signs of active glomerular inflammation in IgAN should likely be addressed early, together with optimal supportive therapy, to prevent progression to chronic morphologic changes and to improve patient prognosis. Of note, due to renal functional reserve, considerable nephron loss can occur before changes to clinical indicators, such as eGFR or serum creatinine levels, become evident [15]. Therefore, fast-acting agents that target inflammation in the glomerulus are expected to produce optimal outcomes.

Agents targeting inflammation that are currently in use for the treatment of patients with IgAN, with variable degrees of evidence, include systemic glucocorticoids, targeted-release budesonide, cytotoxic agents such as cyclophosphamide, antimetabolites including azathioprine and mycophenolate mofetil (MMF), and other agents such as calcineurin inhibitors and hydroxychloroquine [2, 3, 6].

There are several limitations with current inflammation-targeting agents. For example, systemic glucocorticoids, although sometimes effective, are associated with off-target adverse effects and increased risk of infection, requiring reduced dosage, duration and, where appropriate, antimicrobial prophylaxis [2, 6]. Although targeted-release budesonide reduces proteinuria and maintains kidney function during therapy, there appears to be a diminution of effect after stopping medication and, consequently, the optimal duration of treatment is unknown [16]. Azathioprine has also been associated with adverse effects and a lack of treatment effect [2, 6, 17]. Furthermore, use of MMF and hydroxychloroquine are suggested only in China, while cyclophosphamide is reserved for rapidly progressive IgAN [2]. Given these limitations, there is an unmet need for novel agents that more effectively target inflammation in IgAN, with improved tolerability and safety profiles.

With activation of the lectin and alternative complement pathways being a critical factor in driving active glomerular inflammation, agents targeting these pathways could reduce the inflammatory events (after hit 4) in the pathogenesis of IgAN [3]. Importantly, given the continuous overproduction of Gd-IgA1 in patients with IgAN, sustained complement inhibition may be necessary to prevent flares of disease activity. Recent regulatory (Food and Drug Administration) approval of iptacopan (a complement factor B inhibitor) provides a treatment option to limit glomerular inflammation [3, 5]. Several other anti-complement treatments are also currently in development for IgAN. Investigative anti-complement therapies include but are not limited to: other alternative pathway inhibitors targeting factor B or factor D, such as IONIS-FB-L_Rx_ and vemircopan, respectively; and selective inhibitors of terminal pathway components C3, C5 and the C5a receptor, such as pegcetacoplan and ARO-C3, cemdisiran and ravulizumab, and avacopan, respectively [3]. Selective inhibition of the lectin pathway has been explored in IgAN using the mannose-binding lectin-associated serine protease 2 (MASP-2) inhibitor narsoplimab, but the drug failed to produce positive results in the phase 3 ARTEMIS-IGAN trial (NCT03608033) following an outsized placebo effect in the control arm of the study [18]. Histologic evidence indicates the presence of lectin pathway activation in approximately 25%–40% of patients with IgAN, which highlights the importance of patient selection in the evaluation of targeted therapies [13, 19].

Other investigational agents with anti-inflammatory potential include therapies that aim to reduce pathogenic IgA1 production and dampen proximal immune events (hit 1 and 2) [3]. One such approach includes the CD20-targeted B cell–depleting therapy rituximab; however, a randomized controlled trial of rituximab in IgAN failed to reduce serum Gd-IgA1 or anti–Gd-IgA1 antibody levels and did not significantly improve kidney function or proteinuria, despite achieving effective depletion of B cells [20]. Alternative strategies currently under active investigation include the plasma cell–depleting therapies bortezomib, felzartamab and mezagitamab, as well as B-cell modulators targeting B cell–activating factor (BAFF) and/or a proliferation-inducing ligand (APRIL) such as atacicept, telitacicept, povetacicept, sibeprenlimab and zigakibart [3].

Although B cell–targeted therapies aim to address the early events of IgAN pathogenesis by reducing immune complex production, they may not directly target ongoing glomerular inflammation, as residual immune complex deposition may continue to amplify the inflammatory response and cause glomerular damage. In this case, combination approaches incorporating complement inhibition could be more effective in targeting the active glomerular inflammation phenotype. However, as the active inflammatory and chronic fibrotic phenotypes in IgAN are not yet well defined, and therapies have not been differentially investigated in patients with these phenotypes, conclusions regarding the efficacy of specific agents for each phenotype cannot currently be made. Nevertheless, as the treatment landscape for IgAN continues to evolve, appropriate assessment of the active inflammatory and chronic fibrotic phenotypes is likely to play an increasingly critical role in patient management. For instance, integrating clinical assessments with histological insights provided by repeat kidney biopsy may become relevant for guiding treatment decisions or providing a more comprehensive evaluation of treatment responses beyond traditional clinical parameters [21]. In addition, the identification and validation of relevant biomarkers will be crucial for enhancing patient assessment and enabling personalized treatment strategies.

Further research is essential to establish the defining characteristics of active glomerular inflammation in IgAN and, in turn, the most effective therapies for patients with this phenotype. Emerging therapeutic strategies may offer promising avenues to effectively manage active glomerular inflammation in IgAN.
